# The Effects of Interleukin-1*β* in Tumor Necrosis Factor-*α*-Induced Acute Pulmonary Inflammation in Mice

**DOI:** 10.1155/2009/958658

**Published:** 2009-11-04

**Authors:** Sara Saperstein, Heidie Huyck, Elizabeth Kimball, Carl Johnston, Jacob Finkelstein, Gloria Pryhuber

**Affiliations:** ^1^Department of Environmental Medicine, University of Rochester School of Medicine, 601 Elmwood Avenue Box 850, Rochester, NY 14642, USA; ^2^Department of Pediatrics, University of Rochester School of Medicine, 601 Elmwood Avenue Box 850, Rochester, NY 14642, USA

## Abstract

We determined the role of interleukin-1*β* (IL-1*β*) signaling on tumor necrosis factor alpha-induced (TNF-*α*) lung neutrophil influx as well as neutrophil chemoattractant macrophage inflammatory protein (MIP-2) and KC and soluble TNF-*α* receptor (TNFR) levels utilizing wildtype (WT), TNF receptor double knockout (TNFR1/TNFR2 KO), and IL-1*β* KO mice after oropharyngeal instillation with TNF-*α*. A significant increase in neutrophil accumulation in bronchoalveolar lavage fluid (BALF) and lung interstitium was detected in the WT mice six hours after TNF-*α* exposure. This correlated with an increase in BALF MIP-2. In contrast, BALF neutrophil numbers were not increased by TNF-*α* treatment of IL-1*β* KOs, correlating with a failure to induce BALF MIP-2 and a trend toward increased BALF soluble TNFR1. TNF-*α*-instillation increased lavage and serum KC and soluble TNFR2 irrespective of IL-1*β* expression. These results suggest IL-1*β* contributes, in part, to TNF-*α*-mediated, chemokine release, and neutrophil recruitment to the lung, potentially associated with altered soluble TNFR1 release into the BALF.

## 1. Introduction

Inhalation of infectious agents results in an acute pulmonary inflammatory response characterized by the recruitment and accumulation of neutrophils, (polymorphonuclear, PMN) cells, to the lung. This occurs through up-regulation of adhesion molecules and chemotactic signals by various cell types including epithelial, endothelial, and inflammatory cells. Upon migration to the sites of infection or injury, PMNs neutralize pathogens through phagocytosis and the release of superoxide which is converted into microbiocidal hypochlorous acid [1].

The importance of neutrophils in host defense has been shown in many studies. For instance, mice treated with influenza virus strain HK×31 rapidly recruited neutrophils to the upper and lower airways where they played a critical role in limiting virus replication during the early and later phases of infection [2]. However, PMNs also contribute to the pathogenesis of increased pulmonary vascular permeability and lung injury through the release of proteases and oxidants, thereby contributing to the development of acute lung inflammatory diseases [3–6].

The pleiotropic mediators tumor necrosis factor-*α* (TNF-*α*) and interleukin-1*β* (IL-1*β*) are potent cytokines that play an important role in acute and chronic lung inflammatory diseases by inducing the production of various chemokines, growth factors, and adhesion molecules like intercellular adhesion molecule 1 (ICAM-1) [7–9]. TNF-*α* and IL-1*β* are produced by similar cell types including macrophages, PMNs, and epithelial cells. However, TNF-*α* interacts with two distinct receptors, TNFR1 and TNFR2, to transduce its biologic effects whereas IL-1*β* signals through IL-1R1. The two TNF receptors and the IL-1*β* decoy receptor, IL-1 receptor antagonist or IL-1Ra, are proteolytically cleaved from the cell surface by metalloproteases generating soluble forms (sTNFR1, sTNFR2, and soluble IL-1Ra) with the potential to antagonize the bioactivity of the soluble cognate ligands [10–12].

Literature shows a role for IL-1*β* in modulating TNF-*α* bioactivity; for instance, in human pulmonary cells IL-1*β* induced TNFR1 release whereas in human gingival fibroblasts IL-1*β* enhanced sTNFR2 levels without altering shedding of TNFR1. Ex vivo/in vitro studies have shown that IL-1*β* induced the expression of tracheal TNF receptors time dependently [13–17]. Previously we have shown in a pulmonary epithelial type II-like cell line that IL-1*β* enhanced TNF-*α*-mediated expression of the two neutrophil chemoattractants macrophage inflammatory protein-2 (MIP-2) and KC at the transcriptional level in association with increasing surface expression of both TNFRs thereby potentially contributing to the acute inflammatory response induced by TNF-*α* [18].

The impact, however, of IL-1*β* on TNF-*α*-mediated lung inflammation in vivo is incompletely defined. In this study, we utilized wildtype (WT) C57BL/6J and IL-1*β* KO mice to examine the role of the interleukin on acute TNF-*α*-induced interstitial lung neutrophil accumulation and influx as well as alterations in the levels of the two soluble TNF receptors and secretion of MIP-2 and KC.

## 2. Materials and Methods

### 2.1. Wild-Type and Knockout Mice

TNFRsf1a/1b (TNFR1/2) double-null mice were regenerated in a C57BL/6J background. TNFRsf1a^tm1mak/j–/–^ and TNFRsf1b^tm1Mwm/J–/–^ single-knockout (KO) mice [16] were each backcrossed 12 generations onto C57BL/6J and then interbred to produce double-transgenic null mice. Wild-type (WT) C57BL/6J mice were used as experimental controls (The Jackson Laboratory). IL-1*β* ^−/−^ mice generated on a C57BL/6J background were a kind gift from Dr. Yoichiro Iwakura at the University of Tokyo. Mice used in the current protocol were bred and maintained in microisolator cages in specific pathogen-free rooms in the animal care facility at the University of Rochester Medical Center (Rochester, NY). Sentinel animals maintained in the same rooms, on bedding mixed with bedding taken from other cages within the room, routinely tested negative for common murine pathogens, including murine hepatitis, pinworm, and Sendai virus. All animal care and experimental protocols were approved by the University of Rochester Committee on Animal Research and follow the guidelines of IUCAC.

### 2.2. Treatment of Mice

Six-to-ten week-old mice were lightly anesthetized via inhalation of isoflurane, prior to oropharyngeal (OP) instillation (50 *μ*L) of saline as vehicle control or recombinant murine truncated tumor necrosis factor-*α* (rmTNF-*α*, 5 *μ*g/50 *μ*L saline/mouse, R&D Systems, Minneapolis, MN). Six hours after instillation, animals were euthanized by intraperitoneal injection of sodium pentobarbital (5 mg/mouse) followed by exsanguination and pneumothorax.

### 2.3. Analysis of Bronchoalveolar Lavage (BAL) and Cells Isolated from BALF

For BAL fluid collection, the trachea was intubated, the anterior chest wall removed, and the lungs lavaged with normal saline (room temperature, 1 mL × 10). The first two lavage aliquots were centrifuged at 300 × g for 10 minutes at 4°C to pellet the harvested cells. The supernatant was frozen at −80°C until assayed for total protein by bicinchoninic acid assay (BCA, Pierce, Rockford, IL) and for cytokines and receptors. The remaining eight BALF aliquots were combined and centrifuged. The supernatant was discarded. The pelleted BALF cells from all ten aliquots were resuspended in Hanks Balanced Salt Solution** (**HBSS). A total BALF cell count was performed by hemocytometer. BALF cellular differential was determined on 100 *μ*L cytospins stained with Diff-Quik (Dade Behring, Newark, DE). Lung homogenate was prepared in radioimmunoprecipitation assay (RIPA) buffer supplemented with an antiprotease cocktail tablet (1 tablet/10 mLs, Roche Diagnostics, Indianapolis, IN). Total protein content of the homogenates was determined by BCA assay.

### 2.4. Analysis of Soluble TNFR1, Soluble TNFR2, MIP-2, and KC

Lung protein homogenates, serum, and BALF were analyzed by sandwich ELISA (R&D Systems) for soluble TNF receptors, MIP-2, and KC according to manufacturer's instructions. BALF and lung homogenate values were normalized to total protein of the samples assessed by BCA.

### 2.5. Examination of Interstitial Neutrophil Accumulation

The left lung was inflation fixed with 10% buffered formalin overnight, dehydrated to 70% ethanol, and paraffin embedded. Tissue sections were deparaffinized and then sequentially hydrated in passages through xylene, 100% ethanol, 95% ethanol, 70% ethanol, and distilled water. Samples were subjected to antigen retrieval with immersion of slides in 1X Dako Target Retrieval Solution (DAKO, Carpinteria, CA S16990, pH 6.0) and then blocked with 3% hydrogen peroxide. After washing with TRIS-buffered salt containing 0.1% Tween (TBS-T), slides were incubated for 1 hour in TBS-T containing 5% normal mouse serum at room temperature and then further incubated overnight at 4°C in the dark in TBS-T containing rat antimouse neutrophil allotypic marker antibody (1 : 100, Serotec, Raleigh, NC, MCA771G). Next, slides were washed with TBS-T and then incubated with Rat Probe (Rat on Mouse HRP-Polymer kit, catalog number RT517H, Biocare Medical, Concord, CA) for 15 minutes at room temperature. After washing off excess with TBS-T, Rat Polymer-HRP (Biocare Medical) was added for an additional 15 minutes. The slides were rinsed again in TBS-T and staining was performed by the addition of diaminobenzidine chromogen (DAB) substrate (DAB Peroxidase Substrate kit, catalog number DB801L, Biocare Medical) and then counterstained with hematoxylin (Cat Hematoxylin, catalog number CATHE-MM, Biocare Medical) and bluing solution (Bluing reagent, Richard-Allan Scientific, Kalamazoo, MI, 7301) before dehydrating again in 75% ethanol, 95% ethanol, and 100% ethanol and finally xylene. The number of PMNs localized to the lung interstitium was evaluated by MetaMorph software (Molecular Devices, Downingtown, PA) which facilitated counting of four to twelve random photomicrographs (10×) from each experimental lung section; nine to eleven sections were analyzed per experimental group. Interstitial PMNs were counted and then normalized to total number of cells within the field.

### 2.6. TUNEL Stain

Tissue sections were deparaffinized and rehydrated through graded ethanol as described above. Sections were then stained with the terminal deoxynucleotidyl transferase-mediated dUTP nick-end labeling (TUNEL) method using the ApopTag Red In Situ apoptosis detection kit (catalog number S7165, Chemicon International, Billerica, MA) according to the instructions of the manufacturer. Slides were then washed four times (two minutes each) with PBS then visualized by florescence microscopy with a Texas Red filter cube. The TUNEL method labels cells containing fragmented DNA, a hallmark of apoptosis. Positive labeling was achieved by adding the enzyme terminal deoxynucleotidyl transferase (TdT) to catalyze a template-independent addition of nucleotide triphosphates to the 3′-OH ends of fragmented DNA, forming an oligomer composed of digoxigenin. A rhodamine conjugated, antidigoxigenin antibody was then added, nuclei were counterstained with DAPI, and positive-labeled cells were evaluated by examination of tissue sections using epifluoresence at a magnification of 20×. Cells undergoing apoptosis were recognized by an intensely fluorescent nucleus. For quantitative analysis, fluorescent cells were counted in at least 4-5 sections per animal, and mean number of TUNEL-positive (TUNEL+) cells per section was determined. All observations reported are based on analysis of multiple tissue sections from 3 to 12 mice per group.

### 2.7. Statistical Analysis

The results were analyzed by ANOVA followed by Fisher's PLSD and Scheffe's post hoc analyses using StatView software (Acton, MA). All data in this study are expressed as the mean ± SEM, *n* = 8–15 animals per treatment group. A *P* value ≤ .05 was considered significant.

## 3. Results

### 3.1. TNF-*α* Induces an Acute Neutrophilic Response in Wildtype (WT) Mice That Is Deficient in IL-1*β* Knockout (KO) Mice

Oropharyngeal (OP) instillation of recombinant murine truncated tumor necrosis factor-*α* (TNF-*α*, 5 *μ*g/mouse) six hours prior to harvest resulted in a significant reduction in total cell number measured from the bronchoalveolar lavage fluid (BALF) of WT and IL-1*β* KO animals, respectively, compared to saline-treated control mice (Table 1). The reduction was greater in WT than in IL-1*β* KO mice. This reduction was reflected in a decrease in the percentage of the cells that were alveolar macrophages (AMs) in TNF-*α*-treated WT mice as compared to saline-treated WT and TNF-*α*-exposed IL-1*β* KO mice. The absolute number of macrophages in the BALF also trended downward although did not reach statistical significance in either TNF-treated strain. This was not associated with an increase in the number of TUNEL positive cells measured by TUNEL stain (data not shown). In the WT, the decrease in percent macrophages correlated with a significant increase in neutrophils (PMN) as compared to saline WT control mice as well as to TNF-*α*-treated IL-1*β* KO mice, both in terms of percentage and in absolute number (Table 1). There was no increase in BALF neutrophils in TNF-*α*-instilled, as compared to saline-treated IL-1*β* KO mice. Finally, total protein in the BALF was not significantly altered in any treatment group (data not shown). As control for the specificity of the instilled TNF-*α* preparation, there were no significant effects of TNF-*α* treatment in TNF receptor double KO mice for all cellular and protein parameters tested (data not shown). In summary, these data show that IL-1*β* plays a role in the rapid recruitment of neutrophils to the lung during TNF-*α*-mediated pulmonary inflammation.

### 3.2. TNF-*α*-Induced Increase in Interstitial Neutrophils in WT Compared to IL-1*β* KO Mice

**Figure 1:**
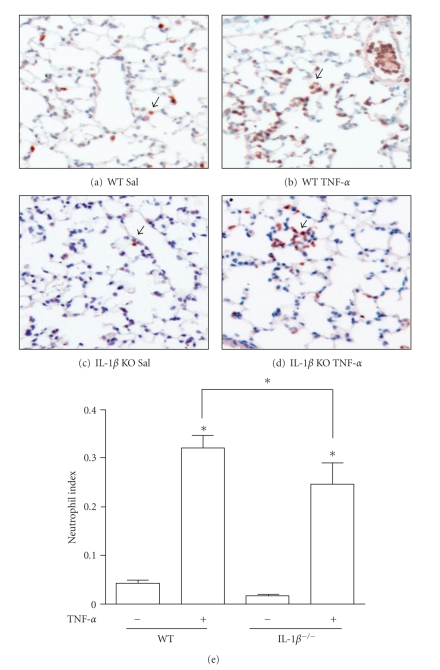
*Neutrophil accumulation in mouse lungs exposed to TNF-*α*.* Wildtype (WT) or IL-1*β* knockout (‒/‒KO) mice were instilled into the oropharynx (OP) with saline (50 *μ*L) or rmTNF-*α* (5 *μ*g in 50 *μ*L saline/mouse). After 6 hours, lungs were formalin fixed and neutrophil antigen-positive cells were identified by immunohistochemistry (arrows). Images were captured through 10x objective. In the graphical analysis, a neutrophil index was determined as the ratio of neutrophil-positive cells per total number of nuclei per low power field. Three to twelve random fields were analyzed per lung section. Data are represented as mean ± SEM, *n* = 9–11 lung sections per TNF group. ∗ represents *P* ≤ .05 versus saline controls or (brackets) between cytokine-treated mice.

TNF-*α* induced a characteristic inflammatory response that included increased neutrophil index, defined as the ratio of neutrophil-antigen positive cells to total number of cell nuclei per low-power field, detected within the lungs of mice regardless of genotype (Figure 1(e)). These neutrophil-antigen positive cells were found throughout the parenchyma in addition to within blood vessels and minimally in large conducting airways (Figures 1(a)–1(d)). However, quantification of the extent of neutrophil accumulation revealed that WT, TNF-*α*-instilled animals had significantly greater recruitment of interstitial PMNs compared to TNF-*α*-treated IL-1*β* KO mice. The average neutrophil index of control, saline-treated WT and IL-1*β* KO mice was 4 ± 3% and 2 ± 1.7% (mean ± SD), respectively, consistent with that seen in normal, untreated animals, as compared to 33% ± 12% and 21% ± 14.7% in TNF-*α*-instilled mice. Neutrophil accumulation was noted within alveolar walls and within vessels of TNF treated mice, more uniformly in WT than in IL-1*β* deficient animals.

### 3.3. Effects of TNF-*α* on Soluble TNF Receptors between WT and IL-1*β* KO Mice

**Figure 2:**
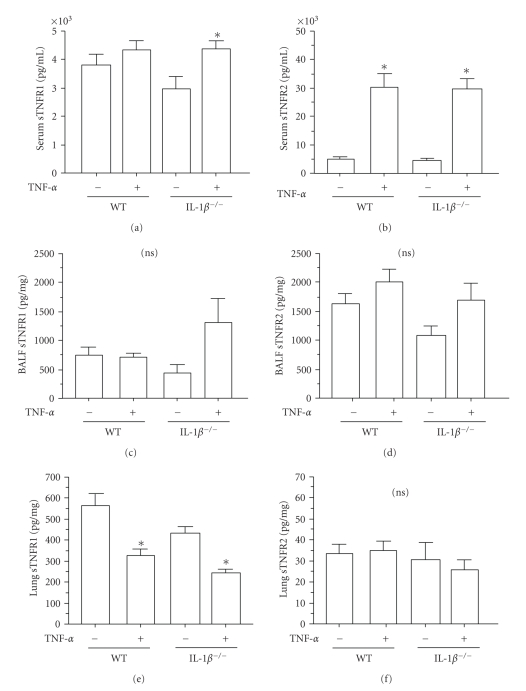
*Detection of soluble receptors in WT and IL-1* *β* *KO mice*. WT or IL-1*β* KO mice were OP instilled with either saline (50 *μ*L) or TNF-*α* (5 *μ*g/50 *μ*L/mouse). After 6 hours, serum, BALF, and lung homogenate were isolated and analyzed for ((a), (c), and (e)) TNFR1 or ((b), (d), and (f)) TNFR2 by ELISA, and for lavage and lung, normalized to protein in samples measured by BCA. Results are mean ± SEM, (*n* = 8–15 mice per group). ∗ represents *P* ≤ .05 saline versus TNF-*α*-treatment of single strain or (brackets) for saline or cytokine-treated comparisons between strains.

TNF receptors are cleaved by metalloproteases from the cell surface to create soluble forms capable of binding TNF-*α* [19, 20]. Previously, it has been shown that IL-1*β* modulates TNF receptor shedding in vitro, thereby impacting cell responsiveness to TNF-*α* [18]. In order to determine the in vivo role of IL-1*β* expression on ligand-induced TNF receptor shedding, measurements of both soluble (sTNFRs) TNFR1 and sTNFR2 were analyzed in lung homogenate, serum, and BALF in response to saline and TNF-*α*. As expected, sTNFRs were not detectable in TNF receptor double KO animals (data not shown). Interestingly, serum, BALF, and lung sTNFR1 concentrations tended to be lower in the saline treated IL-1*β* null mice as compared to WT (Figures 2(a), 2(c), 2(e)). Following TNF-*α* treatment, serum sTNFR1 levels were either slightly or significantly increased in WT and IL-1*β* KO animals, respectively, although there were no differences between TNF-*α*-exposed animals. BALF sTNFR1 levels tended to be increased in IL-1*β* KO animals compared to both saline controls and WT and TNF-*α*-treated mice though this did not reach statistical significance. Interestingly, BALF sTNFR1 levels were unchanged from control in TNF-*α* treated WT animals (Figure 2(c)). Similar to our previous studies in a lung epithelial cell line in vitro [18]*,* both transgenic mice had a significant decrease in lung sTNFR1 after TNF-*α* exposure, although there were no differences between TNF-*α*-exposed animals (Figure 2(e)). While there were no differences in lung or BALF sTNFR2 between treatment or genotype, sTNFR2 was markedly increased in the serum of both animal strains treated with TNF-*α* as compared to saline controls (Figures 2(b), 2(d), 2(f)). Together these data suggest that while IL-1*β* expression does not impact TNF-*α*-mediated sTNFR2, the interleukin may play a negative role in the release of serum and BALF-derived, ligand-mediated TNFR1.

### 3.4. Reduced BALF MIP-2 from TNF-*α*-Exposed IL-1*β* KO Mice Compared to WT Animals

**Figure 3:**
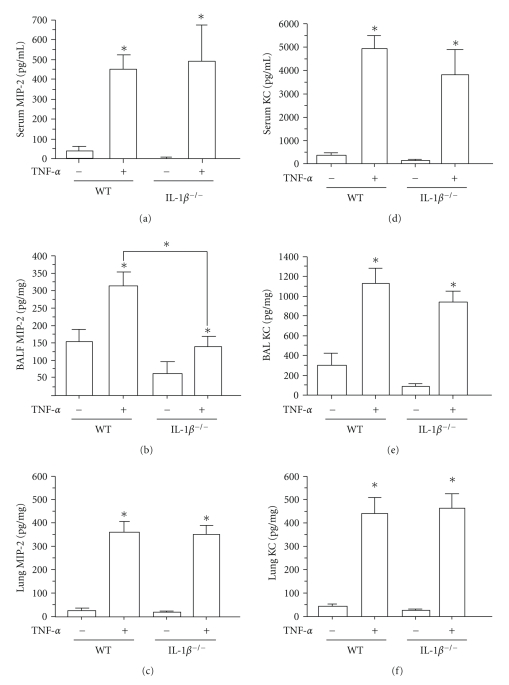
*Analysis of MIP-2 after TNF-*α* instillation*. WT or IL-1*β* KO mice were OP instilled with either saline (50 *μ*L) or TNF-*α* (5 *μ*g/50 *μ*L/mouse). After 6 hours, serum, BALF, and lung homogenate were isolated and analyzed for ((a), (b), and (c)) MIP-2 and ((d), (e), and (f)) by ELISA. BALF and lung homogenate values are normalized to protein content measured by BCA. Results are mean ± SEM, (*n* = 8–15). ∗ represents *P* ≤ .05 saline versus TNF-*α*-treatment of single strain or (brackets) for saline or cytokine-treated comparisons between strains.

The two neutrophil chemoattractant proteins MIP-2 and KC are induced by both TNF-*α* and IL-1*β* in response to inflammatory stimuli such as silica or infection [21]. In order to determine whether IL-1*β*-mediated alterations in PMN recruitment and interstitial accumulation correlated with alterations in TNF-*α*-mediated chemokine production, MIP-2 and KC were evaluated from the BALF, serum, and lungs. As shown in Figure 3(a), serum MIP-2 was significantly increased in both TNF-*α*-treated IL-1*β* KO and WT animals. However, BALF-derived MIP-2 was only significantly increased in TNF-*α*-instilled WT mice while BALF MIP-2 from TNF-*α*-treated IL-1*β* KO animals was not greater than saline-treated controls and was depressed relative to WT, suggesting that IL-1*β* plays a role in TNF-*α*-induced production of MIP-2 found in the BALF (Figure 3(b)). Finally, although markedly increased compared to saline controls, lung MIP-2 levels were no different between transgenic animals treated with TNF-*α* (Figure 3(c)). As shown in Figures 3(d)–3(f), serum, BALF, and lung KC content markedly increased after TNF-*α* treatment compared to saline controls but reached levels that were not different between WT or IL-1*β* KO animals.

## 4. Discussion

Tumor necrosis factor-*α* (TNF-*α*) is a pleiotropic cytokine essential for lung immune and inflammatory responses to microbial challenge [22, 23]. Inappropriate induction or sustained activation of TNF-*α* signaling has been linked to the development of acute and chronic lung disorders characterized by the activation of resident cells and subsequent migration and accumulation of immune cells including macrophages and neutrophils [8, 24–26]. Due to the consistent association between neutrophils and lung injury in humans and animal models, and the propensity of neutrophils and their products to cause tissue injury in experimental systems, it has been theorized that PMNs play an important role in the development of acute lung injury (ALI) [25, 26].

TNF-*α* mediates inflammatory cell recruitment in part by inducing the expression of chemokines including macrophage inflammatory protein-2 (MIP-2) and KC by activating two unique receptors, TNFR1 and TNFR2. MIP-2 and KC are potent neutrophil chemoattractants that belong to the same (CXC) family of chemokines and that are produced in the mouse at sites of tissue inflammation after infection or injury [27, 28]. We recently reported, in a murine lung epithelial cell line, that TNF-*α* up-regulates surface expression and shedding of TNFR2 while down-regulating TNFR1 in association with enhanced mRNA expression and secretion of MIP-2 and KC [18].

However, in addition to TNF-*α*, early high levels of another proinflammatory cytokine, interleukin-1*β* (IL-1*β*) have been found in murine models of ALI [17, 18]. IL-1*β* shares many of the biological properties of TNF-*α*, including induction of chemokine expression, and studies have shown that it also modulates shedding and mRNA expression of TNF receptors [13, 19]. In murine lung epithelial cells, IL-1*β* enhanced TNF-*α*-mediated MIP2 and KC by increasing surface expression of lung epithelial TNF receptors [18]. Based on these observations, the present study investigated the hypothesis that IL-1*β* enhances TNF-*α*-induced neutrophil recruitment to the lung by altering TNF receptors as well as MIP-2 and KC production.

We found, in wildtype (WT) C57Bl/6J mice that oropharyngeal instillation with TNF-*α* enhanced the percentage and absolute number of PMNs in the lavage fluid (BALF) and within the interstitium which corresponded with a decrease in the percentage of BALF alveolar macrophages as well as total lavage cell number. TNF-*α* also mediates cell death processes in addition to its inflammatory function in immune cells; therefore this was regarded as a potential mechanism behind the decrease in BALF cell number. TUNEL staining was performed to assess apoptosis; however TNF-*α* exposure did not detectably enhance TUNEL positive-stained cells within the lungs of animals. Further analysis specifically of lavage-derived cells, including activation and adherence to or transmigration through airway epithelium, may aid in elucidating mechanisms behind the TNF-*α*-mediated acute decrease in BALF cell number.

In accordance with previous findings, TNFR1 was decreased in the TNF-*α*-treated lungs while sTNFR2 was enhanced in lavage (trend) and serum correlating with an increase in BALF, serum, and lung MIP-2 and KC [29]. These findings are consistent with the response of lung epithelial cell lines to TNF-*α* in vitro [18]. However, we also found that in contrast with our recent findings, lung TNFR2 levels were not altered between saline and TNF-*α*-treated WT mice, while lavage sTNFR1 tended to be increased in the TNF-*α*-treated WT [18]. The discrepancies between in vivo and in vitro systems may be due to several factors. First, the numerous cell types in the airway accessible to lavage as well as in the lung parenchyma, including macrophages, potentially regulate TNF receptor shedding in response to ligand differentially from the epithelial cells studied in vitro. In addition, sTNF receptors may accumulate in culture media whereas mice have the ability to mobilize shed receptors into the circulation where they can be eliminated via urination [30]. Likewise, serum soluble receptor content, which is the summation of receptor release from numerous cell types in various organ systems, is balanced by renal clearance and thus may not reflect as well solubilization occurring in the airway. In addition, the relatively low TNFR2 content detected in the lung tissue homogenates may suggest that despite, or perhaps due to, detergents in the lysis buffer, the receptor in these samples may not have been readily available to the assay system and content may be under-estimated. Interestingly, in the process of this study it was noted that addition of some preparations of bovine serum albumin to TNFR2 standards prior to ELISA masked the protein from detection (data not shown). The dilution of serum and BALF samples prior to analysis appeared to overcome this inhibition.

With regards to IL-1*β* KO mice, loss of interleukin expression resulted in a trend towards constitutively reduced BALF, lung, and serum sTNFR1 compared to WT mice. BALF and serum MIP-2 were also slightly lower in KO mice instilled with saline. Further study may uncover a role for IL-1*β* in basal TNFR1 expression. Investigation into the role of IL-1*β* in TNF-*α*-mediated events demonstrated that IL-1*β* KO animals had a deficient neutrophilic (PMN) inflammatory response, as measured in both BALF and in lung interstitium, after TNF-*α* instillation which correlated with a reduction in BALF-derived macrophage inflammatory protein-2 (MIP-2) measured at the same time point. No clear evidence for a role of IL-1*β* in TNF-*α*-induced shedding of soluble TNF receptors was detected in this study. Overall, these findings are consistent with previous studies which demonstrated that IL-1*β* is critical for pulmonary inflammation and fibrosis [31–34]. However, it is noted that the loss of IL-1*β* was insufficient to prevent the TNF dependent induction of BALF, serum, and lung-derived KC as well as serum and lung-MIP-2.

It is not clear why the lack of IL-1*β* reduced TNF-mediated accumulation of MIP-2 and neutrophils in BALF. Perhaps the TNF-*α*-induced activation of interstitial PMNs and macrophages in either genotype contributed to the increase in lung-MIP-2 whereas airway cells are responsible for lavagable MIP-2 and transmigration of neutrophils. Likewise, parenchymal cells may have played a role in up-regulating KC in BALF and lung, which was not affected by loss of IL-1*β*. To more specifically address cell type specific response origins in vivo*,* experiments utilizing chimeric mice in which IL-1*β* or its receptor, IL-1RI, is present only on parenchymal or inflammatory cells, such as by irradiating and then transplanting animals with bone marrow of reciprocal transgenic mice, could further aid in interpreting the up-regulation of individual chemokines after TNF-*α* stimulation. For instance, Pryhuber et al. demonstrated that parenchymal cell TNF receptor expression was key to inflammatory cell recruitment and respiratory failure in *Pneumocystis carinii*-induced pneumonia [35]. In addition, differential temporal regulation of each chemokine has also been demonstrated. Studies inoculating mice with *Legionella pneumophila* showed that levels of KC peaked one day earlier than MIP-2 while still other investigators have found overlapping production of each chemokine in models of inflammation [36–38]. It is possible that the time-point selected for the present study misses IL-1*β*-modification of TNF-*α*-induced KC.

It is also possible that the interleukin-mediated contribution to TNF-*α* cytokine production is redundant. For example, the related IL-1*α* may enhance the induction of KC found in the BALF and lungs of both WT and IL-1*β* null mice. Studies have shown that IL-1*α* plays a role in stabilizing KC mRNA, primarily through IRAK1 [39–41]. Moreover, Son and Roby showed, in granulosa cells isolated from immature mice at 28 days of age, that exposure to IL-1*α* induced KC mRNA and protein [42]. Studies of TNF-*α*-induced lung inflammatory response in IL-1 receptor deficient mice or in the presence of IL-1RI antagonists may clarify the role of both IL-1*α* and *β*.

Inhibition of interleukin signaling has been shown to be efficient in treating several destructive diseases such as systemic onset juvenile idiopathic arthritis, hereditary periodic fever syndromes, and gout arthritis [43–46]. Our results, showing the role of IL-1*β* in modifying TNF-*α*-induced pulmonary inflammation, are in support of a consideration for IL-1*β* neutralization in treatment of acute, and potentially chronic, TNF-*α* mediated lung diseases. Therefore, in pulmonary diseases characterized by neutrophil accumulation and excessive inflammation, loss of IL-1*β* bioactivity may be useful in modulating pulmonary inflammatory diseases, potentially without increasing the risk of serious adverse secondary effects such as pulmonary infections which are seen in rheumatoid arthritis (RA) and Crohn's disease (CD) patients using anti-TNF-*α* therapeutic agents.

## Figures and Tables

**Table 1:** Cell count and differential of bronchoalveolar lavage harvested six hours after oropharyngeal delivery of TNF alpha or control saline. BAL: bronchoalveolar lavage; Lymphs: lymphocytes. Treatment groups represent 8–15 mice/group.*P ≤ .05 TNF versus saline, C57BL6/J treatment groups; ^‡^ *P* ≤ .05 IT TNF versus Saline, IL*β* ^−/−^ treatment groups; ^#^ *P* ≤ .05 IT TNF, IL-1*β* ^−/−^ versus WT treatment groups. Values are means ± SEM,*n* = 8 − 15 mice.

	Cell type						
Group	BAL Cell Count (×10^4^/mL)	% Macrophages	% Neutrophils	% Lymphs	Macrophage # (×10^4^/mL)	Neutrophil # (×10^4^/mL)	Lymph # (×10^4^/mL)
Saline, C57BL6/J	41.3 ± 6.2	96.0 ± 1.2	2.1 ± 0.6	1.9 ± 0.9	39.9 ± 6.2	0.86 ± 0.3	0.57 ± 0.3
TNF, C57BL6/J	26.0 ± 2.8*	89.7 ± 2.0*	8.5 ± 2.0*	1.9 ± 0.7	29.7 ± 5.9	2.23 ± 0.4*	0.65 ± 0.3
Saline, IL-1*β* ^−/−^	57.0 ± 7.2	97.7 ± 0.8	1.5 ± 0.7	0.8 ± 0.3	56.0 ± 7.4	0.66 ± 0.3	0.30 ± 0.1
TNF, IL-1*β* ^−/−^	39.8 ± 5.4^‡#^	97.0 ± 0.8^#^	1.6 ± 0.5^#^	1.4 ± 0.6	38.7 ± 5.4	0.65 ± 0.3^#^	0.46 ± 0.2

## References

[1] Downey DG, Bell SC, Elborn JS (2009). Neutrophils in cystic fibrosis. *Thorax*.

[2] Tate MD, Brooks AG, Reading PC (2008). The role of neutrophils in the upper and lower respiratory tract during influenza virus infection of mice. *Respiratory Research*.

[3] Matthay MA, Zimmerman GA (2005). Acute lung injury and the acute respiratory distress syndrome: four decades of inquiry into pathogenesis and rational management. *American Journal of Respiratory Cell and Molecular Biology*.

[4] Lo SK, Everitt J, Gu J (1992). Tumor necrosis factor mediates experimental pulmonary edema by ICAM-1 and CD18-dependent mechanisms. *Journal of Clinical Investigation*.

[5] Lindbom L (2003). Regulation of vascular permeability by neutrophils in acute inflammation. *Chemical Immunology and Allergy*.

[6] Hu G, Vogel SM, Schwartz DE (2008). Intercellular adhesion molecule-1-dependent neutrophil adhesion to endothelial cells induces caveolae-mediated pulmonary vascular hyperpermeability. *Circulation Research*.

[7] Piguet PF, Collart MA, Grau GE (1990). Requirement of tumour necrosis factor for development of silica-induced pulmonary fibrosis. *Nature*.

[8] Ortiz LA, Lasky J, Hamilton RF (1998). Expression of the TNF and the necessity of TNF receptors in bleomycin-induced lung injury in mice. *Experimental Lung Research*.

[9] Sakurada S, Kato T, Okamoto T (1996). Induction of cytokines and ICAM-1 by proinflammatory cytokines in primary rheumatoid synovial fibroblasts and inhibition by N-acetyl-L-cysteine and aspirin. *International Immunology*.

[10] Xanthoulea S, Pasparakis M, Kousteni S (2004). Tumor necrosis factor (TNF) receptor shedding controls thresholds of innate immune activation that balance opposing TNF functions in infectious and inflammatory diseases. *The Journal of Experimental Medicine*.

[11] van Mierlo GJD, Scherer HU, Hameetman M (2008). Cutting edge: TNFR-shedding by CD4 + CD25 + regulatory T cells inhibits the induction of inflammatory mediators. *Journal of Immunology*.

[12] Cui X, Rouhani FN, Hawari F, Levine SJ (2003). Shedding of the type II IL-1 decoy receptor requires a multifunctional aminopeptidase, aminopeptidase regulator of TNF receptor type 1 shedding. *Journal of Immunology*.

[13] Cardell LO, Uddman R, Zhang Y (2008). Interleukin-1*β* up-regulates tumor necrosis factor receptors in the mouse airways. *Pulmonary Pharmacology and Therapeutics*.

[14] Lukacs NW, Strieter RM, Chensue SW (1995). TNF-*α* mediates recruitment of neutrophils and eosinophils during airway inflammation. *Journal of Immunology*.

[15] Gater PR, Wasserman MA, Paciorek PM (1996). Inhibition of Sephadex-induced Lung Injury in the Rat by Ro 45-2081, a Tumor Necrosis Factor Receptor Fusion Protein. *American Journal of Respiratory Cell and Molecular Biology*.

[16] Piguet PF, Vesin C (1994). Treatment by human recombinant soluble TNF receptor of pulmonary fibrosis induced by bleomycin or silica in mice. *European Respiratory Journal*.

[17] Moreland LW, Baumgartner SW, Schiff MH (1997). Treatment of rheumatoid arthritis with a recombinant human tumor necrosis factor receptor (p75)-Fc fusion protein. *New England Journal of Medicine*.

[18] Saperstein S, Chen L, Oakes D (2009). IL-1*β* Augments TNF-*α*-mediated inflammatory responses from lung epithelial cells. *Journal of Interferon & Cytokine Research*.

[19] Mullberg J, Durie FH, Otten-Evans C (1995). A metalloprotease inhibitor blocks shedding of the IL-6 receptor and the p60 TNF receptor. *Journal of Immunology*.

[20] Reddy P, Slack JL, Davis R (2000). Functional analysis of the domain structure of tumor necrosis factor-*α* converting enzyme. *The Journal of Biological Chemistry*.

[21] Pryhuber GS, Huyck HL, Baggs R (2003). Induction of chemokines by low-dose intratracheal silica is reduced in TNFR I (p55) null mice. *Toxicological Sciences*.

[22] Strieter RM, Kunkel SL (1994). Acute lung injury: the role of cytokines in the elicitation of neutrophils. *Journal of Investigative Medicine*.

[23] Wajant H, Pfizenmaier K, Scheurich P (2003). Tumor necrosis factor signaling. *Cell Death and Differentiation*.

[24] Gilroy DW, Lawrence T, Perretti M (2004). Inflammatory resolution: new opportunities for drug discovery. *Nature Reviews Drug Discovery*.

[25] Abraham E, Carmody A, Shenkar R (2000). Neutrophils as early immunologic effectors in hemorrhage- or endotoxemia-induced acute lung injury. *American Journal of Physiology - Lung Cellular and Molecular Physiology*.

[26] Fujishima S, Aikawa N (1995). Neutrophil mediated tissue injury and its modulation. *Intensive Care Medicine*.

[27] Huang S, Paulauskis JD, Godleski JJ, Kobzik L (1992). Expression of macrophage inflammatory protein-2 and KC mRNA in pulmonary inflammation. *American Journal of Pathology*.

[28] Rovai LE, Herschman HR, Smith JB (1998). The murine neutrophil-chemoattractant chemokines LIX, KC, and MIP-2 have distinct induction kinetics, tissue distributions, and tissue-specific sensitivities to glucocorticoid regulation in endotoxemia. *Journal of Leukocyte Biology*.

[29] Higuchi M, Aggarwal BB (1994). TNF induces internalization of the p60 receptor and shedding of the p80 receptor. *Journal of Immunology*.

[30] Novick D, Engelmann H, Wallach D (1989). Soluble cytokine receptors are present in normal human urine. *Journal of Experimental Medicine*.

[31] Raines EW, Dower SK, Ross R (1989). Interleukin-1 mitogenic activity for fibroblasts and smooth muscle cells is due to PDGF-AA. *Science*.

[32] Dinarello CA (1997). Interleukin-1. *Cytokine & Growth Factor Reviews*.

[33] Kolb M, Margetts PJ, Anthony DC (2001). Transient expression of IL-1*β*
induces acute lung injury and chronic repair leading to pulmonary fibrosis. *The Journal of Clinical Investigation*.

[34] Gasse P, Mary C, Guenon I (2007). IL-1R1/MyD88 signaling and the inflammasome are essential in pulmonary inflammation and fibrosis in mice. *The Journal of Clinical Investigation*.

[35] Pryhuber GS, Huyck HL, Bhagwat S (2008). Parenchymal cell TNF receptors contribute to inflammatory cell recruitment and respiratory failure in Pneumocystis carinii-induced pneumonia. *Journal of Immunology*.

[36] Tateda K, Moore TA, Newstead MW (2001). Chemokine-dependent neutrophil recruitment in a murine model of Legionella pneumonia: potential role of neutrophils as immunoregulatory cells. *Infection and Immunity*.

[37] Call DR, Nemzek JA, Ebong SJ (2001). Differential local and systemic regulation of the murine chemokines KC and MIP2. *Shock*.

[38] Mercer-Jones MA, Shrotri MS, Peyton JC (1999). Neutrophil sequestration in liver and lung is differentially regulated by C-X-C chemokines during experimental peritonitis. *Inflammation*.

[39] Tebo JM, Datta S, Kishore R (2000). Interleukin-1-mediated stabilization of mouse KC mRNA depends on sequences in both 5′- and 3′-untranslated regions. *Journal of Biological Chemistry*.

[40] Hartupee J, Li X, Hamilton T (2008). Interleukin 1*α*-induced NF*κ*B activation and chemokine mRNA stabilization diverge at IRAK. *Journal of Biological Chemistry*.

[41] Datta S, Novotny M, Li X (2004). Toll IL-1 receptors differ in their ability to promote the stabilization of adenosine and uridine-rich elements containing mRNA. *The Journal of Immunology*.

[42] Son DS, Roby KF (2006). Interleukin-1*α*-induced chemokines in mouse granulosa cells: impact on keratinocyte chemoattractant chemokine, a CXC subfamily. *Molecular Endocrinology*.

[43] Hawkins PN, Lachmann HJ, Aganna E (2004). Spectrum of clinical features in muckle-wells syndrome and response to anakinra. *Arthritis and Rheumatism*.

[44] Hoffman HM, Rosengren S, Boyle DL (2004). Prevention of cold-associated acute inflammation in familial cold autoinflammatory syndrome by interleukin-1 receptor antagonist. *Lancet*.

[45] Pascual V, Allantaz F, Arce E (2005). Role of interleukin-1 (IL-1) in the pathogenesis of systemic onset juvenile idiopathic arthritis and clinical response to IL-1 blockade. *The Journal of Experimental Medicine*.

[46] Belkhir R, Moulonguet-Doleris L, Hachulla E (2007). Treatment of familial mediterranean fever with anakinra. *Annals of Internal Medicine*.

